# Government-Expert Joint Intervention with Treatment Algorithm and Improved Hypertension Management and Reduced Stroke Mortality in a Primary-Care Setting

**DOI:** 10.1155/2021/9661576

**Published:** 2021-10-14

**Authors:** Mulalibieke Heizhati, Nanfang Li, Delian Zhang, Suofeiya Abulikemu, Guijuan Chang, Jing Hong, Nuerguli Maimaiti, Junli Hu, Lei Wang, Gulinuer Duiyimuhan

**Affiliations:** Hypertension Center of People's Hospital of Xinjiang Uygur Autonomous Region, Xinjiang Hypertension Institute, National Health Committee Key Laboratory of Hypertension Clinical Research China, No. 91 Tianchi Road, Urumqi 830001, Xinjiang, China

## Abstract

Hypertension management is suboptimal in the primary-care setting of developing countries, where the burden of both hypertension and cardiovascular disease is huge. Therefore, we conducted a government-expert joint intervention in a resource-constrained primary setting of Emin, China, between 2014 and 2016, to improve hypertension management and reduce hypertension-related hospitalization and mortality. Primary-care providers were trained on treatment algorithm and physicians for specialized management. Public education was delivered by various ways including door-to-door screening. Program effectiveness was evaluated using screening data by comparing hypertension awareness, treatment, and control rates and by comparing hypertension-related hospitalization and total cardiovascular disease (CVD) and stroke mortality at each phase. As results, 313 primary-health providers were trained to use the algorithm and 3 physicians attended specialist training. 1/3 of locals (49490 of 133376) were screened. Compared to the early phase, hypertension awareness improved by 9.3% (58% vs. 64%), treatment by 11.4% (39% vs. 44%), and control rates by 33% (10% vs. 15%). The proportion of case/all-cause hospitalization was reduced by 35% (4.02% vs. 2.60%) for CVD and by 17% (3.72% vs. 3.10%) for stroke. The proportion of stroke/all-cause death was reduced by 46% (21.9% in 2011–2013 vs. 15.0% in 2014–2016). At the control area, the proportion of case/all-cause mortality showed no reduction. In conclusion, government-expert joint intervention with introducing treatment algorithm may improve hypertension control and decrease related hospitalization and stroke mortality in underresourced settings.

## 1. Introduction

About 80% of mortality from cardiovascular disease (CVD) occurs in low- and middle-income countries [1], mainly due to the increased prevalence of risk factors including elevated blood pressure (BP) and due to relatively lacking access to medical care in these underresourced countries [2]. Hypertension is one of the major risk factors and key drivers for CVD in these countries, especially in underdeveloped regions, whereas it remains undetected, undertreated, and poorly controlled [3, 4].

Based on nationwide data, prevalence and mortality rates for ischemic cerebrovascular disease, ischemic heart disease, and hypertensive heart disease in Xinjiang, an underdeveloped province located in Northwestern China, are higher than the national average [5, 6]. Hypertension is responsible for >70% of stroke burden in China [6, 7]. In Xinjiang, hypertension is affecting 35.0–40.7% of adults aged ≥35 years [8, 9] and 52.6% of some population [8], higher than national average (41.9%) [10], whereas the treatment (28.8% vs. 34.4%) and control rates (10.9% vs. 15.3%) are lower [8, 10], as in a recent nationwide hypertension survey. It showed that control of hypertension in population aged ≥18 years from less-developed regions including Xinjiang is about 8% [3]. Furthermore, patient-, provider-, and system-related bottlenecks for hypertension control widely exist here [11, 12]. Furthermore, other risk factors for CVD are highly prevalent such as dyslipidemia, obesity, and high salt intake [13].

Although hypertension in developing countries has been studied substantially, a number of critical problems, especially its proper management, remain unsolved [3]. Given the fact that hypertension plays significant roles in CVD morbidity and mortality [5, 6], it is highly needed to undertake interventions to improve hypertension control and to reduce its related morbidity and mortality. Indeed, interventions have been associated with reduction in CVD risk [4, 14]. In the Kaiser program, the control rate of hypertension increased from 44% to 90% and death from stroke fell to 42% over 13 years of the project [15]. While model interventions provide useful experiences and basis, the ability to deliver satisfactory care can be highly contextualized and there is a lack of knowledge about how to stimulate this kind care in low- and middle-income countries. Therefore, we selected Emin county, a multiethnic low-income region with constricted medical resource and >80% population living in the agricultural and stock-raising region characterized by low education attainment (Table 1), to implement a government-expert joint intervention covering the whole county, involving multiple stakeholders, emphasizing education of primary-care providers and physicians and of the public by various ways such as door-to-door screening, and introducing antihypertensive treatment algorithm between Jan 2014 and Dec 2016. The aim was to raise awareness, treatment and control of hypertension, and to decrease hypertension-related hospitalization and mortality including CVD and stroke at a primary-care setting, and therefore, to explore suitable solutions for areas with similar conditions. In the current report, we evaluated the effects of the hypertension control program after two years.

## 2. Methods

### 2.1. Context

Intervention area: Emin, in Northern Xinjiang, is home to about 133376 people aged ≥15 years (45% Han and 35% Kazakh). There were 1 county-level hospital and 22 community health service centers and clinics, with 12 licensed doctors (Table 1).

Control area: Tuoli, a neighbouring county with similar condition about 60 Km away from the Emin county center, was selected as the control area. Adult population aged ≥15 years in Tuoli is about 83106 and comprised of Han (25.3%) and Kazakh (71.4%) ethnic groups. There were 1 county hospital, 1 maternity and child-care hospital, and 1 community health service center with 7 stations. Importantly, there were no documented antihypertension programs in Tuoli during this period.

Study protocols were approved by the ethics committee of People's Hospital of Xinjiang Uygur Autonomous Region, China.

### 2.2. Intervention Design

This is a government-expert joint intervention, involving multiple stakeholders. Depending on previous interventions [15–18] and on local conditions, administration from the top was engaged [10, 11]. The local government was involved by distributing an official statement and by establishing a cross-sectoral steering committee, efforts were orchestrated from health workers and general public, and input from a central hypertension management team was encompassed.

#### 2.2.1. Concrete Methods Included

**Figure 1:**
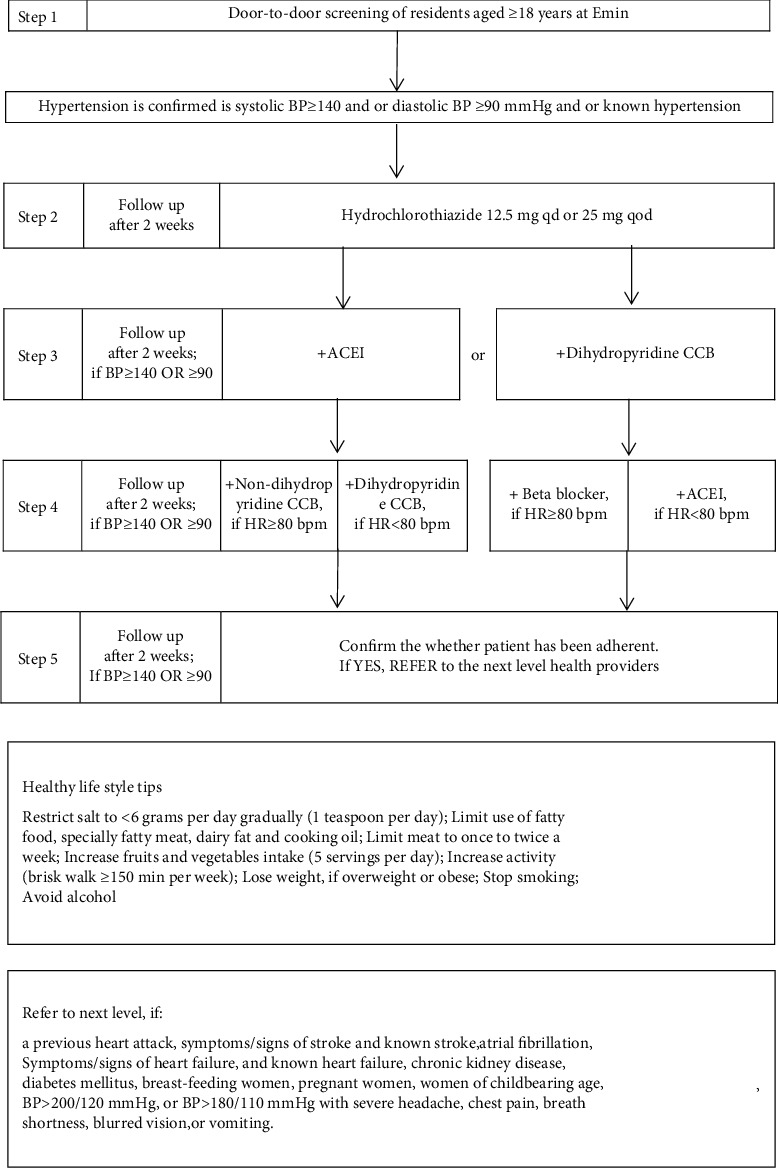
Hypertension treatment algorithm.

(1) Hypertension was set as a priority by distributing an official statement and by establishing a cross-sectoral steering committee; (2) medical staff education; (3) an evidence-based antihypertensive treatment algorithm was introduced to help health-care providers (Figure 1); (4) raising public awareness through various ways including door-to-door screening and public education programs; and (5) contextualizing some parts: materials including books, printed algorithm, and slices were prepared, and training was prepared in 4 languages (Han, Uygur, Kazakh, and Mongolian); considering the cultural and linguistic background, local trusted teachers and hypertensive volunteers delivered health-related information to the general public.

### 2.3. Education of Primary Health-Care Providers and Physicians

Primary health-care providers including general practitioners and physicians were trained at a 3-day workshop at the start and at a refresh 1-day workshop once 3 months later in 4 languages. Training concentrated on treatment algorithm including components on follow-up guided by algorithm and BP and counselling on healthy life style (Figure 1). Physicians from county-level hospitals were also sent to the hypertension center in the regional tertiary hospital to participate in the training on diagnosis of complicated conditions associated with hypertension to help the referred patients from primary care.

### 2.4. Public Education

Public education was undertaken together with door-to-door screening by health providers, by well-planned media (television, newspapers, radio, videos, etc.) and communication messages with a broad range of community activities, involvement of health professionals, volunteers' education (hypertensives and teachers), work site education, gatherings, and handbooks. Contests were conducted regularly at work sites, communities, and among hypertensives as in previous interventions [19].

### 2.5. Introduction of Treatment Algorithm

Gaps exist between evidence-based recommendations and current practice on hypertension management in developing regions, that health-care providers are less informed or experienced and/or lack willingness to follow guidelines, and or that medications may not be used at the best dosage. Therefore, we introduced treatment algorithm.

### 2.6. Rationale for the Treatment Algorithm and Agent Selection

As in Figure 1, we introduced an antihypertensive treatment algorithm with integrative blood pressure (BP) control mechanisms in mind including reduction of sodium and water retention by diuretics, renin aldosterone angiotensin system (RAAS) inhibition by angiotensin-converting enzyme inhibitors (ACEIs), vasodialation by dihydropyridine calcium channel blockers (CCBs), and controlling cardiac rate and output by nondihydropyridine CCB and beta-blockers. We had introduced this algorithm to another three counties of Xinjiang between 1997 and 2017 and observed plausible changes in hypertension management and improvement in related death (just accepted in the International Journal of hypertension). In fact, drug regimens with complementary activity, where a second agent blocks compensatory responses to the initial agent or affects a different pressor mechanism, can result in additive BP lowering [20]. For example, diuretics may stimulate RAAS, and an additive BP-lowering effect may be obtained by adding an ACEI [21]. In addition, to help accelerate clinical decision making, one specific agent (hydroclorothiazide, captopril, nitrendipine, verapamil, and metoprolol) was selected as in the previous program.

### 2.7. Application of the Treatment Algorithm

Step 1: definition of hypertension: systolic BP (SBP) ≥140 and or diastolic BP (DBP) ≥90 mmHg and or known hypertension. Step 2: prescribing hydrochlorothiazide 12.5 mg qd or 25 mg qod and follow-up in 2 weeks. Step 3: adding ACEI or dihydropyridine CCB for another 2 weeks, if BP ≥ 140/90 mmHg. Step 4: if BP ≥ 140/90 mmHg, for the coming 2 weeks, (1) a nondihydropyridine CCB is added if pulse rate ≥80bpm and dihydropyridine CCB if pulse rate <80 bpm for those on ACEI; (2) a beta-blocker is added if pulse rate ≥80 bpm and an ACEI if pulse rate <80 bpm for those on a dihydropyridine CCB. Step 5: referral to county hospital is recommended if BP ≥ 140/90 mmHg after step 4. If BP reached the target level at any phase, follow-up at every 3 months is conducted.

The patients are referred to the next-level hospital if there is a previous heart attack, atrial fibrillation, symptoms/signs of heart failure and known heart failure, chronic kidney disease, symptoms/signs of stroke, known stroke, diabetes, breast-feeding women, pregnant women, women of childbearing age, or BP > 200/120 mmHg and BP > 180/110 mmHg with severe headache, chest pain, breath shortness, blurred vision, or vomiting.

Health-care providers were encouraged to follow the algorithm at the population screening and at their regular clinical work unless clinical discretion required for the abovementioned conditions, since in settings where people do not regularly visit the doctor, people who are just recommended only lifestyle modification may not return for reevaluation and needed treatment, resulting in uncontrolled hypertension and associated complications [22]. For managing known hypertension, health-care providers were advised to review medication and BP; simplify regimens; or change to the antihypertensive treatment algorithm of this program. Recommended target BP was <140/90 mmHg.

Health-care providers were also educated to provide information to patients on the importance of long-term medication intake, even if there are no symptoms, on how to take medications at home, to explain how many times, and at what time to take medication; explain potential adverse effects; and what to do if patients experience.

### 2.8. Door-to-Door Screening

Health workers performed door-to-door screening and public education to eligible residents since 1 Jan 2014 to 31 Dec 2016. The eligible population included locals aged ≥15 years. Informed consent was obtained from all subjects and from the parents or legal guardians for those under 18 years of age.

### 2.9. Data Collection

In Emin, anthropometric and clinical measurements using questionnaires were taken during door-to-door screening. Questions assessed demographic characteristics, education, lifestyle risk factors (cigarette and alcohol use), and hypertension-related information. Physical examination including BP measurement was conducted as well. We measured BP three times using an electronic sphygmomanometer (Omron HEM-7201) from the unclothed right arm of the person in a sitting position at intervals of at least 1 min after a rest of at least 5 min. Mean of the three BP was used in this analysis. Body weight, height, and waist circumference were measured using standard methods. Body mass index (BMI) was calculated as body weight divided by squared height (kg/m^2^).

In addition, we collected data on hospitalization and hospital stay length for hypertension, CVD, and stroke from the medical insurance system and county hospitals both in the intervention and control area between 2014 and 2016. Furthermore, we collected death data (death diagnosis and certificate) from the hospital information and household registration system of both the intervention and control area between 2011 and 2016. However, we did not collect BP- and hypertension-related data from the population in the control area.

### 2.10. Grouping and Evaluation

To evaluate the overall impact of the intervention, first, we divided study participants into three groups as early-, middle-, and later-phase intervention groups, based on the time of being screened (numbered by the screening date and tertiled), and compared the awareness, treatment, and control of hypertension and BP levels among participants in early-, middle-, and later-phase groups. Second, we compared the proportion of case/all-cause hospitalization and hospital stay length for hypertension, CVD, and stroke among participants in early-, middle-, and later-phase groups, using the main diagnosis at hospital discharge. Third, we compared the proportion of case/all-cause death for CVD and stroke using death diagnosis on the death certificate from the hospital information and household registration system at the intervention (Emin) and control (Tuoli) area before (2011–2013) and during the intervention (2014–2016).

### 2.11. Statistical Analysis

Continuous data such as age, BMI, waist circumference, and SBP and DBP were expressed as mean ± SD if normally distributed and compared using the ANOVA test followed by the post hoc analysis (LSD) test among the groups. Categorical data including gender composition, ethnic groups, education attainments, living area, and smoking and drinking status were expressed as (*n*,%) and compared using the *X*^2^ test among the groups where necessary. Prevalence, awareness, treatment, and control rates of hypertension were calculated, expressed as %, 95% confidence interval, and compared using the *X*^2^ test among groups. The proportion of case/all-cause hospitalization and of case/all-cause hospital stay for hypertension, CVD, and stroke and the proportion of case/all-cause mortality for CVD and stroke were calculated, expressed as %, and compared using the *X*^2^ test among the groups. SPSS software version 19 was used for statistical analysis, and *P* < 0.05 was considered statistically significant.

## 3. Results

### 3.1. Activity Metrics

During the intervention, 313 health-care providers were trained for the algorithm once 3 months and 3 physicians attended training at the regional hypertension center for 3 months each time.

### 3.2. Statistics for Screening Subjects

During the door-to-door visit, 59405 subjects were counseled on healthy life and invited for screening, of whom 49497 subjects (response rate: 83.3%) were screened, covering 37.1% (49497/133376) of adult residents, and there were complete data for 47040 subjects.

The early-, middle-, and later-phase subjects were similar in age and gender composition, whereas the later-phase subjects were less smokers and drinkers and had lower BMI and waist circumference (Table 2).

Compared to the early phase, awareness, treatment, and control rates improved by 9.3% (early vs. later phase: 58% vs. 64%), 11.4% (39% vs. 44%), and 33% (10% vs. 15%). The control rate of treated hypertension improved by 31.9% (20.4 vs. 27%) (Table 3).

Compared to the early phase, total hypertensives (146.9 vs. 144.4 vs. 144.1 mmHg) and hypertensives with awareness (145.2 vs. 143.9 vs. 143.4 mmHg) and under treatment (143.9 vs. 141.7 vs. 141.5 mmHg) at the middle and later phase showed significant lower SBP levels.

In addition, total population and total hypertensives showed lower DBP levels at the later phase, compared to the early phase (77.4 vs. 76.7 mmHg and 89.3 vs. 87.8 mmHg, respectively, Table 4).

### 3.3. Statistics on Hypertension-Related Hospitalization and Death Data

Compared to the early phase, the proportion of case/all-cause hospitalization reduced by 35.3% (4.02% vs. 2.60%) for total CVD and by 16.6% (3.72% vs. 3.10%) for stroke (Table 5). The proportion of case/total hospital stay reduced by 32.5% (4.34 vs. 2.93%) for CVD and by 10.8% (4.46 vs. 3.98%) for stroke (Table 5).

Compared to 2011–2013, the proportion of total CVD/all-cause death and of stroke/all-cause death showed a decreasing trend in 2014–2016 at Emin county. The proportion of CVD/all-cause death reduced by 17% (33% vs. 27.4%, statistically nonsignificant), and that of stroke/all-cause death reduced by 31.5% (21.9% vs. 15.0%, *P* < 0.001) in 2014–2016.

In the control area, the proportion of total CVD and stroke/all-cause death showed a minor but not significant decrease during the same period (Table 6).

## 4. Discussion

Hypertension is a key CVD driver and remains widely undetected, undertreated, and poorly controlled, especially in underdeveloped areas [3, 4]. This is a government-expert joint program with tranings of health-care providers and introduction of an algorithm. Main results encompass awareness, treatment, and control rates for hypertension improved by 9.3%, 11.4%, and 33% at the later phase than the early phase. The proportion of case/all-cause hospitalization reduced by 17.3% for hypertension, by 35% for CVD, and by 17% for stroke. The proportion of stroke/all-cause death was reduced by 31.5%.

At the early phase, >40% of hypertensives remain unaware, >60% untreated, and >90% BP uncontrolled, possibly indicating major bottlenecks for ineffective control are diagnosis, effective screening, and treatment titration. Hence, screening and quality improvement to ensure titration of medications may be critical for better outcomes [18]. After 2 years, awareness reached 64%, higher than national average (46.9%) [3]. Raising awareness is the first step for successful hypertension management, and the better way is screening [4]. Door-to-door screening and public education may have benefited the population by increasing health awareness [23]. Treatment and control rates are 44% and 15% at the later phase, comparable to the national average (40.7% and 15.3%) [3] and those of many parts of the world (13%) [24]. One important aspect may be the involvement of the local government. Another critical part possibly is the algorithm as put forward in the WHO HEARTS program [22]. Its presence may have helped clinical decision making by addressing the difficulty in choosing medicine [10, 11]. Additionally, an agent was recommended from each type, which may have facilitated agent prescription.

At the later phase, the program achieved some success in hypertension management, reflected by improved treatment and control rates, and by the decreased hypertension-related hospitalization and hospital stay. In fact, better out-of-hospital BP control has a positive effect on reducing hospitalization [25–28]. The proportion of stroke/all-cause death during the intervention was reduced by 31.5%. The program may have helped capacity building on hypertension management, followed by decreased stroke mortality through better out-of-hospital BP control, reduction in stroke incidence, and improvement in its survival [29]. While it is difficult to provide a comprehensive stroke mortality analysis in regional programs, we have tried to explore this by including the data with comparable regions. Unlike Emin, the hypertension program was not conducted at Tuoli, where the death proportion for CVD including stroke was not changed much. Although these data may not establish causality, an expected pattern of correlation between the program and stroke mortality composition is observed in the intervention county.

Prevalence, awareness, treatment, and control of hypertension were 26%, 58%, 39%, and 10% at the start. Prevalence and awareness were higher than the national average (23.2% and 46.9%) [3], possibly driven by the difference in survey methods. The population with health problems is more prone to accept screening and have increased health concerns, which may have some relevance with higher prevalence and awareness. Also, Kazakhs and Mongolians with higher hypertension prevalence [8, 9, 30] account for 39% of the study population. Control rate at the early phase (10%) was comparable to the 8% for less-developed areas in the national data [3] and comparable to the national average at the later phase (15.0% vs. 15.3%) [3]. Improvements have been more ideal if individuals combated hypertension through lifestyle and followed up [31].

Current analysis is strengthened by subjects covering over one-third of locals and by their diversity in age, sex, and ethnicity, which may make them representative of locals [32]. Nonetheless, several points deserve consideration when interpreting the data. First, the before-after comparison design does not allow us to establish causal relation between the program and observed plausible changes. Results would have been more ideal if the same subjects were included, whereas a cross-sectional approach is a common way [33]. In addition, it was aimed to raise public awareness. Second, effects of counselling on healthy life style and patient compliance were not evaluated. However, beneficial changes in BP might suggest the success in part. Furthermore, lack of a control area should also be kept in mind in terms of secular trends. Nevertheless, we addressed this element by comparing changes in the proportion of CVD and stroke observed in Emin county to the neighboring Tuoli county. In addition, results just after the 2-year time may not be affected by the secular trends, based on similar examples [34]. Finally, we failed to collect the data on the average time to reach the target BP and the number of agents taken by the patients at the time of target BP, which are the important indicators to assess the efficiency and effectiveness of an algorithm. However, previous studies from community-based patients of China reported that treating hypertension with nitrendipine and hydrochlorothiazide and with nitrendipine and metoprolol will allow more reasonable and efficient allocation of limited resources in low-income countries [35].

In conclusion, government-expert joint intervention with the introduction of the treatment algorithm may help capacity building on hypertension management and decrease hypertension-related CVD morbidity and mortality at a medical resource-constricted primary setting with low income and education.

## Figures and Tables

**Table 1:** Population characteristics at Emin and Tuoli Xinjiang China.

Characteristics	Emin	Tuoli
Population aged ≥15 years (*n*)	133376	83106
Ethnicity (*n*, %): Han	60019 (45.0)	21025 (25.3)
Kazakh	46681 (35.0)	59337 (71.4)
Uygur	6669 (5.0)	1072 (1.29)
Mongolian	5335 (4.0)	—
Others	14671 (11.0)	1662 (2.0)
Education (*n*, %): no schooling	3614 (2.71)	2493 (3.0)
Primary	46148 (34.6)	27424 (33.0)
Junior high	5551 (41.5)	37397 (45.0)
Senior high	17739 (13.3)	9972 (12.0)
College or above	16271 (12.2)	5817 (7.0)
Region (*n*, %), urban	44814 (33.6)	29087 (35.0)
Agricultural and stock-raising	10889 (81.4)	54019 (65.0)
No. of doctors for per 1000 population and of those with license	3.1 and 12	2.4 and 10
Medical insurance coverage (%)	>90% of population	
Gross domestic product in 2014	Xinjiang: ranking 25^th^ among 31 provinces	

**Table 2:** Characteristics of the population screened at the early, middle, and later phase.

Characteristics	Early phase	Middle phase	Later phase	*P*1	*P*2	*P*3	*P*
Total (*n*)	15680	15680	15680				
Gender (men, *n*, %)	8052 (51.4)	11295 (48.5)	8157 (52.0)	—	—	—	0.015
Age (years)	43.6 ± 15.1	44.7 ± 15.5	43.4 ± 15.4	<0.001	0.289	<0.001	<0.001
Body mass index (kg/m^2^)	24.6 ± 3.4	24.6 ± 3.4	24.4 ± 3.5	—	0.028	0.016	<0.001
Waist circumference (cm)	85.1 ± 11.5	85.3 ± 12.4	84.8 ± 11.6	—	0.002	<0.001	<0.001
Smoking status (*n*, %)	2321 (14.8)	3242 (13.9)	1902 (12.1)	—	—	—	0.002
Drinking status (*n*, %)	2965 (18.9)	4287 (18.4)	2458 (15.7)	—	—	—	<0.001

*P*1: early vs. middle phase; *P*2: early vs. later phase; *P*3: middle vs. later phase; *P*: for among-group comparison.

**Table 3:** Awareness, treatment, and control rates of hypertension at the early, middle, and later phase (%, 95% CI).

	Early phase	Middle phase	Later phase	*X* ^2^/*P*
Total subjects (*n*)	15680	15680	15680	
Hypertensive patients (*n*)	4030	4265	4615	
Age-standardized prevalence	25.9 (25.2, 26.6)	27.2 (26.3, 28.1)	29.3 (29.2, 30.3)	54.65/<0.001
Awareness of hypertension	58.0 (56.0, 59.0)	66.7 (66.1, 68.0)	64.0 (63.3, 66.1)	32.67/<0.001
Treatment of hypertension	39.0 (38.1, 41.0)	46.1 (45.0, 47.5)	44.2 (43.1, 45.2)	22.14/<0.001
Treatment of in the aware	67.5 (65.2, 69.1)	68.0 (67.2, 69.2)	68.6 (67.0–70.0)	2.34/0.834
Control of hypertension	10.0 (9.1, 11.0)	17.1 (16.2, 18.5)	15.0 (14.1, 16.2)	48.52/<0.001
Control of treated hypertension	20.4 (19.3, 21.3)	29.4 (29.1, 31.5)	27.0 (26.5, 28.4)	30.32/<0.001

95% CI: 95% confidence interval.

**Table 4:** Blood pressure levels in study participants at the early, middle, and later phase.

	Early phase	Middle phase	Later phase	*P*1	*P*2	*P*3	*P*
Systolic blood pressure (mmHg)							
Total	123.8 ± 17.60	121.9 ± 16.8	123.2 ± 15.4	<0.001	—	<0.001	
Hypertensives	146.9 ± 16.1	144.4 ± 13.4	144.1 ± 11.1	<0.001	<0.001	0.047	<0.001
Aware	145.2 ± 14.4	143.9 ± 12.3	143.4 ± 10.7	<0.001	<0.001	0.046	<0.001
Unaware	145.9 ± 10.5	145.1 ± 14.5	146.4 ± 8.5	—	—	—	—
Treated	143.9 ± 16.1	141.7 ± 11.7	141.5 ± 14.9	<0.001	<0.001	—	<0.001
Untreated	147.8 ± 12.1	147.1 ± 17.7	148.1 ± 12.7	—	—	—	—
Controlled	131.4 ± 6.7	131.4 ± 7.1	132.2 ± 6.4	—	—	—	—
Uncontrolled	144.6 ± 12.5	146.1 ± 16.1	144.6 ± 12.0	<0.001	—	<0.001	

Diastolic blood pressure (mmHg)							
Total	77.4 ± 10.3	76.6 ± 9.8	76.7 ± 9.1	—	—	—	—
Hypertensives	89.3 ± 9.7	89.2 ± 6.9	87.8 ± 8.5	—	<0.001	<0.001	<0.001
Aware	88.1 ± 7.6	87.9 ± 10.3	88.0 ± 9.3	<0.001	0.793	<0.001	<0.001
Unaware	80.3 ± 7.8	80.9 ± 9.5	84.5 ± 5.3	—	<0.001	<0.001	<0.001
Treated	88.4 ± 8.4	88.4 ± 9.5	89.3 ± 7.4	—	—	—	—
Untreated	84.2 ± 9.1	85.2 ± 11.1	86.5 ± 6.7	—	—	<0.001	—
Controlled	81.6 ± 6.0	81.5 ± 6.4	82.9 ± 4.7	—	—	—	—
Uncontrolled	86.3 ± 9.2	88.4 ± 10.4	88.5 ± 7.3	<0.001	<0.001	—	<0.001

*P*1: early vs. middle phase; *P*2: early vs. later phase; *P*3: middle vs. later phase; *P*: among-group comparison.

**Table 5:** Changes in the proportion of case/total hospitalization and of case hospital stay days for hypertension, CVD, and stroke at the early, middle, and later phase at the intervention area.

	Early phase	Middle phase	Later phase	*P*
All-cause				
Subjects (*n*)	18258	18122	21083	
Total hospital stay (days)	165023	161084	185519	

Hypertension				
Subjects (*n*)	1852	1586	1769	
Total hospital stay (days)	17800	15012	16974	
Average hospital stay (days)	9.61	9.47	9.60	
% of case/total hospitalization	10.14	8.75	8.39	<0.001
% of case/total hospital stay	10.79	9.32	9.15	<0.001

Total CVD				
Subjects (*n*)	735	663	548	
Total hospital stay (days)	77169	6492	5430	
Average hospital stay (days)	9.75	9.79	9.91	
% of case/total hospitalization	4.02	3.65	2.60	<0.001
% of case/total hospital stay	4.34	4.03	2.93	<0.001

Total stroke				
Subjects (*n*)	680	624	654	
Total hospital stay (days)	7361	6661	7390	
Average hospital stay (days)	10.83	10.68	11.30	
% of case/total hospitalization	3.72	3.44	3.10	<0.001
% of case/total hospital stay	4.46	4.14	3.98	<0.001

CVD: cardiovascular disease.

**Table 6:** Changes in the proportion of case/all-cause death for total CVD and stroke in intervention and control before and during the intervention period.

	2011–2013	2014–2016	*X* ^2^/*P*
Emin (intervention area)			
All-cause death (*n*)	1258	1298	
Total CVD death (*n*)	415	355	
Proportion of CVD in all-cause death (%)	33.0	27.4	1.14/0.684
Stroke death (*n*)	276	195	
Proportion of stroke in all-cause death (%)	21.9	15.0	16.78/<0.001

Tuoli (control area)			
All-cause death (*n*)	1554	2004	
Total CVD death (*n*)	533	653	
Proportion of CVD in all-cause death (%)	34.3	32.6	1.87/0.188
Stroke death (*n*)	347	435	
Proportion of stroke in all-cause death (%)	22.3	21.7	0.001/.0997

CVD: cardiovascular disease.

## Data Availability

Data can be made available on reasonable request.

## References

[1] (2011). *Global Status Report on Noncommunicable Diseases 2010*.

[2] Yusuf S., Rangarajan S., Teo K. (2014). Cardiovascular risk and events in 17 low-, middle-, and high-income countries. *New England Journal of Medicine*.

[3] Wang Z., Chen Z., Zhang L. (2018). Status of hypertension in China. *Circulation*.

[4] (2013). *A Global Brief on Hypertension*.

[5] Zhou M., Wang H., Zhu J. (2016). Cause-specific mortality for 240 causes in China during 1990–2013: a systematic subnational analysis for the global burden of disease study 2013. *The Lancet*.

[6] Wang W., Jiang B., Sun H. (2017). Prevalence, incidence, and mortality of stroke in China. *Circulation*.

[7] Liu M., Wu B., Wang W.-Z., Lee L.-M., Zhang S.-H., Kong L.-Z. (2007). Stroke in China: epidemiology, prevention, and management strategies. *The Lancet Neurology*.

[8] Wang Y.-T., Adi D., Yu Z.-X. (2017). The burden and correlates of hypertension among Chinese rural population in Han, Uygur, and Kazak: a cross-sectional study. *Journal of the American Society of Hypertension*.

[9] Jiang J., Zhang B., Zhang M. (2015). Prevalence of conventional cardiovascular disease risk factors among Chinese Kazakh individuals of diverse occupational backgrounds in Xinjiang China. *International Journal of Cardiology*.

[10] Li W., Gu H., Teo K. K. (2016). Hypertension prevalence, awareness, treatment, and control in 115 rural and urban communities involving 47 000 people from China. *Journal of Hypertension*.

[11] Basu S., Millett C. (2013). Social epidemiology of hypertension in middle-income countries. *Hypertension*.

[12] MacMahon S., Alderman M. H., Lindholm L. H., Liu L., Sanchez R. A., Seedat Y. K. (2008). Blood-pressure-related disease is a global health priority. *The Lancet*.

[13] Li N., Wang H., Yan Z., Yao X., Hong J., Zhou L. (2012). Ethnic disparities in the clustering of risk factors for cardiovascular disease among the Kazakh, Uygur, Mongolian and Han populations of Xinjiang: a cross-sectional study. *BMC Public Health*.

[14] World Health Organization Executive Board (2013). *Draft Action Plan for the Prevention and Control of Noncommunicable Diseases 2013–2020.11 January 2013*.

[15] Jaffe M. G., Young J. D. (2016). The Kaiser permanente Northern California story: improving hypertension control from 44% to 90% in 13 Years (2000 to 2013). *Journal of Clinical Hypertension*.

[16] Mozheyko M., Eregin S., Danilenko N. (2017). Hypertension in Russia: changes observed after 4 years of a comprehensive health system improvement program in the yaroslavl region. *The Journal of Clinical Hypertension*.

[17] Nissinen A., Berrios X., Puska P. (2001). Community-based noncommunicable disease interventions: lessons from developed countries for developing ones. *Bulletin of the World Health Organization*.

[18] Oti S. O., Van De Vijver S., Gomez G. B. (2016). Outcomes and costs of implementing a community-based intervention for hypertension in an urban slum in Kenya. *Bulletin of the World Health Organization*.

[19] O’Loughlin J. L., Paradis G., Gray-Donald K., Renaud L. (1999). The impact of a community-based heart disease prevention program in a low-income, inner-city neighborhood. *American Journal of Public Health*.

[20] Gradman A. H., Basile J. N., Carter B. L., Bakris G. L. (2011). Combination therapy in hypertension. *The Journal of Clinical Hypertension*.

[21] Whelton P. K., Carey R. M., Aronow W. S. (2018). 2017ACC/AHA/AAPA/ABC/ACPM/AGS/APhA/ASH/ASPC/NMA/PCNA guideline for the prevention, detection, evaluation, and management of high blood pressure in adults. *Hypertension*.

[22] (2018). *Hearts Technical Package for Cardiovascular Disease Management in Primary Health Care: Healthy-Lifestyle Counselling*.

[23] Jaffe M. G., Lee G. A., Young J. D., Sidney S., Go A. S. (2013). Improved blood pressure control associated with a large-scale hypertension program. *JAMA*.

[24] Chow C. K., Teo K. K., Rangarajan S. (2013). Prevalence, awareness, treatment, and control of hypertension in rural and urban communities in high-, middle-, and low-income countries. *Journal of the American Medical Association*.

[25] Yiannakopoulou E. C., Papadopulos J. S., Cokkinos D. V., Mountokalakis T. D. (2005). Adherence to antihypertensive treatment: a critical factor for blood pressure control. *European Journal of Cardiovascular Prevention and Rehabilitation: Official Journal of the European Society of Cardiology, Working Groups on Epidemiology & Prevention and Cardiac Rehabilitation and Exercise Physiology*.

[26] Elliott W. J. (2009). Improving outcomes in hypertensive patients: focus on adherence and persistence with antihypertensive therapy. *The Journal of Clinical Hypertension*.

[27] Breekveldt-Postma N. S., Penning-van Beest F. J. A., Siiskonen S. J. (2008). Effect of persistent use of antihypertensives on blood pressure goal attainment. *Current Medical Research and Opinion*.

[28] Sokol M. C., McGuigan K. A., Verbrugge R. R., Epstein R. S. (2005). Impact of medication adherence on hospitalization risk and healthcare cost. *Medical Care*.

[29] Hata J., Ninomiya T., Hirakawa Y. (2013). Secular trends in cardiovascular disease and its risk factors in Japanese. *Circulation*.

[30] Yan R., Li W., Yin L., Wang Y., Bo J. (2017). Cardiovascular diseases and risk-factor burden in urban and rural communities in high-, middle-, and low-income regions of China: a large community-based epidemiological study. *Journal of the American Heart Association*.

[31] Li D., Lv J., Liu F. (2015). Hypertension burden and control in mainland China: analysis of nationwide data 2003–2012. *International Journal of Cardiology*.

[32] Yeh R. W., Sidney S., Chandra M., Sorel M., Selby J. V., Go A. S. (2010). Population trends in the incidence and outcomes of acute myocardial infarction. *New England Journal of Medicine*.

[33] Cífková R., Škodová Z., Bruthans J. (2010). Longitudinal trends in major cardiovascular risk factors in the Czech population between 1985 and 2007/8. Czech MONICA and Czech post-MONICA. *Atherosclerosis*.

[34] Sarrafzadegan N., Kelishadi R., Esmaillzadeh A. (2009). Do lifestyle interventions work in developing countries? findings from the Isfahan healthy heart program in the Islamic Republic of Iran. *Bulletin of the World Health Organization*.

[35] Wang Z., Chen Z., Wang X. (2017). Cost-effectiveness of nitrendipine and hydrochlorothiazide or metoprolol to treat hypertension in rural community health centers in China. *Journal of Hypertension*.

